# Fisetin Attenuates D-Gal-Induced Ovarian Aging by Modulating Mitophagy via the AMPK/mTOR Pathway

**DOI:** 10.3390/antiox15050602

**Published:** 2026-05-09

**Authors:** Juan Dong, Yaxin Zhu, Zongyang Li, Yanyan Chang, Xiyuan Yan, Caiqiao Zhang, Dong Niu

**Affiliations:** 1 Key Laboratory of Applied Technology on Green-Eco-Healthy Animal Husbandry of Zhejiang Province, Provincial Engineering Research Center for Animal Health Diagnostics & Advanced Technology, Zhejiang International Science and Technology Cooperation Base for Veterinary Medicine and Health Management, China Australia Joint Laboratory for Animal Health Big Data Analytics, College of Animal Science and Technology & College of Veterinary Medicine, Zhejiang A&F University, No. 666, Wusu Road, Hangzhou 311300, China; 20230088@zafu.edu.cn (J.D.); 2024108022036@stu.zafu.edu.cn (Y.Z.); 1601599@stu.zafu.edu.cn (Z.L.); yychang@stu.zafu.edu.cn (Y.C.); 202201170224@stu.zafu.edu.cn (X.Y.); 2 College of Animal Sciences, Zhejiang University, No. 866 Yuhangtang Road, Hangzhou 310058, China

**Keywords:** fisetin, mitophagy, oxidative stress, AMPK/mTOR, ovarian aging

## Abstract

This study aimed to explore the alleviating effects of fisetin, a polyphenolic flavonoid, on ovarian dysfunction in a D-galactose (D-gal)-induced aging mouse model, as well as the underlying mechanisms, using both in vivo and in vitro experiments. Mice were subcutaneously injected with D-gal (100 mg/kg/day) for 60 days to establish the ovarian aging model; during the final 30 days, fisetin (10, 20, 30 mg/kg/day) was given orally. In addition, a senescent model of granulosa cell (GC) was established using D-gal and treated with fisetin. Fisetin supplementation improved ovarian endocrine function and reproductive capacity in aging mice, as reflected by regularized estrous cycles, elevated estradiol levels, and increased embryo numbers. Furthermore, fisetin reduced the number of atretic follicles and the extent of ovarian fibrosis and senescence, while simultaneously restoring the proliferation-apoptosis balance in follicular GCs, as well as alleviating oxidative stress. RNA-sequencing revealed that AMP-activated protein kinase (AMPK)/mechanistic target of rapamycin (mTOR) signaling and mitophagy were involved in the protective effects of fisetin against ovarian aging. Consistently, fisetin treatment promoted mitophagy, accompanied by AMPK/mTOR activation in ovarian tissues and GCs following D-gal exposure. Inhibition of AMPK attenuated the effect of fisetin on mitophagy. Additionally, blockage of mitophagy also reversed the beneficial effects of fisetin on mitochondrial injury, oxidative stress, cell cycle arrest, and cellular senescence in D-gal-induced senescent GCs. These findings indicate that fisetin prevents ovarian aging by suppressing follicular GC oxidative damage and ameliorating cell cycle arrest via activation of AMPK/mTOR-mediated mitophagy, thereby preserving female fertility.

## 1. Introduction

The ovary is one of the earliest organs to undergo functional failure in females, characterized by a marked reduction in follicular number and oocyte quality, eventually resulting in infertility, spontaneous abortion, and birth defects. The rate of ovarian follicular atresia increases with advancing maternal age, mainly due to diminished function of follicular GCs [1]. In follicles, GCs act as the supporting and nourishing cells, facilitating the transfer of energy substances, hormones, and signals to oocytes via gap junctions, thereby determining follicular fate in terms of activation, growth, or atresia. Oxidative stress, arising from an imbalance between reactive oxygen species (ROS) overproduction and reduced antioxidant enzyme activity such as total superoxide dismutase (T-SOD) and catalase (CAT), has been recognized as a major promoter of follicular atresia, largely attributed to the attack of ROS on GCs [2]. High levels of ROS cause severe mitochondrial dysfunction in follicular GCs by directly attacking membrane lipids, proteins, and mtDNA, leading to loss of membrane potential, impaired oxidative phosphorylation, reduced adenosine triphosphate (ATP) synthesis, and ultimately cell cycle arrest [3]. Furthermore, damaged mitochondria release pro-apoptotic factors such as cytochrome C, activate the caspase cascade, and induce GCs apoptosis, thereby accelerating follicular atresia [4]. Thus, targeting oxidative stress-induced mitochondrial injury in GCs is a promising approach to improve cell survival, counteracting the age-related loss of follicular quality.

Mitophagy is a selective autophagic process that specifically degrades damaged mitochondria within cells, thereby reducing ROS accumulation and maintaining mitochondrial homeostasis. The PTEN-induced putative kinase 1 (PINK1)-Parkin pathway serves an essential function in controlling mitophagy. Upon mitochondrial damage, PINK1 accumulates on the outer mitochondrial membrane, recruiting and activating Parkin to induce ubiquitination of mitochondrial proteins, which are then recognized by SQSTM1 (p62) and bind to LC3 (microtubule-associated protein 1 light chain 3) to form autophagosomes [5]. Recent studies have confirmed that under energy stress, mitophagy plays a critical role in follicular growth and supports GC survival by removing damaged mitochondria or other mitochondria-derived apoptogenic factors. For instance, PINK1-Parkin-mediated mitophagy could decrease the Bcl-2-associated X (Bax)/B cell lymphoma 2 (Bcl-2) ratio, inhibit cleaved caspase-3 expression, and restore mitochondrial integrity, thereby alleviating porcine GC injury under hypoxic stress [6]. Similarly, in bovine ovarian GCs, activation of mitophagy significantly reduced the elevated apoptosis rate and ROS levels, which in turn further protected GCs against oxidative stress-induced damage [7]. Cisplatin treatment has been shown to induce ovarian dysfunction in women, as evidenced by increased GC apoptosis, ROS elevation, ATP depletion, and loss of mitochondrial membrane potential, all of which are alleviated by mitophagy activation, a key cellular self-protection mechanism [8]. Collectively, these findings point to targeting mitophagy as an effective therapeutic strategy to mitigate age-related oxidative damage to mitochondria in follicular GCs and delay the decline of ovarian function.

Fisetin (3,3′,4′,7-tetrahydroxyflavone) is a bioactive flavonoid abundantly present in a wide range of fruits and vegetables [9]. Several studies have revealed that fisetin has multiple pharmacological functions, including antioxidant, anti-inflammatory, and anti-apoptotic effects, as well as a favorable safety profile [10,11]. Given its antioxidant properties, the action of fisetin in treating ovarian disorders has attracted considerable research interest. For instance, high-dose fisetin supplementation significantly protected ovarian function from oxidative stress in a rat polycystic ovary syndrome model by enhancing glutathione (GSH) levels and SOD activity [12]. Additionally, oral administration of fisetin mitigated ovarian ischemia–reperfusion injury in rats, an effect associated with elevated levels of the antioxidants T-SOD, CAT, and GSH and reduced malondialdehyde (MDA) production [13]. However, the potential mechanism of fisetin involved in delaying ovarian aging is still unknown. Recently, increasing research has revealed that fisetin promotes cell survival by activating mitophagy, which prevents the accumulation of damaged mitochondria and the excessive production of mitochondrial ROS. For instance, fisetin administration induced mitophagy in cerebral microvascular endothelial cells, a process that scavenged ROS and blocked inflammatory activation within the central nervous system of mice [14]. In addition, fisetin injection in rats triggered PINK1-Parkin-mediated mitophagy in periodontal tissues, which played a key role in clearing damaged mitochondria and alleviating oxidative stress associated with periodontitis [15]. Therefore, these findings suggest that mitophagy mediates the protective effect of fisetin against cellular oxidative damage, but whether this mechanism also underlies the ability of fisetin to prevent ovarian aging remains unclear.

Here, D-gal was used to establish a mouse aging model in vivo and to induce GC senescence in vitro. D-gal is a reducing sugar that, at high doses, induces aging in animals and cells both in vivo and in vitro, closely mimicking natural aging, and is therefore widely used to investigate the mechanisms of aging and to evaluate the anti-aging efficacy of various compounds [16,17]. Mechanistically, D-gal triggers cellular senescence through multiple pathways, including excessive ROS production and impaired mitochondrial function, ultimately leading to cell cycle arrest [18]. Nevertheless, different from the D-gal-induced accelerated aging model, natural aging results from the gradual, decades-long accumulation of genetic, environmental, and metabolic factors and involves multiple parallel mechanisms including telomere attrition, genomic instability, epigenetic drift, and mitochondrial dysfunction, most of which are largely irreversible [19]. Therefore, the rapid induction of age-like phenotypes within weeks cannot recapitulate the progressive follicular depletion and cumulative DNA damage that occur over decades in the female ovary, limiting the assessment of long-term intervention effects and indicating that results from D-gal-based models should be extrapolated to natural aging with caution. This study aims to investigate the attenuating effects of fisetin against ovarian aging in D-gal-induced mice, as well as the underlying molecular mechanisms, which provides knowledge that supports the future potential of fisetin for delaying ovarian aging in female, through further validation in natural aging models is warranted.

## 2. Materials and Methods

### 2.1. Animals and Treatments

100 female BALB/c mice (6-week-old, 16.5 ± 0.5 g) were purchased from Charles River Laboratories Animals Ltd. (Beijing, China). Mice were housed five per cage under a 12 h light/dark cycle (lights on 08:00–20:00) with free access to food and water, in a controlled environment (23 ± 1 °C, 55 ± 5% humidity). All animal procedures were performed in strict accordance with international ethical standards for animal experiments and were approved by the Institutional Animal Care and Use Committee of Zhejiang A&F University, China (ZAFUAC2023005). At 1-week following acclimatization, mice were randomly assigned to five groups (n = 20): Control, D-gal, D-gal+fisetin (10 mg/kg), D-gal+fisetin (20 mg/kg), and D-gal+fisetin (30 mg/kg). D-gal (MB1853-2, Meilunbio, Dalian, China) was dissolved in phosphate-buffered saline (PBS), whereas fisetin (MB5836, Meilunbio) was dissolved in a vehicle prepared by mixing 10% dimethylsulfoxide (DMSO), 40% PEG 300, 5% Tween 80, and 45% PBS. Mice in the D-gal treatment groups received a daily subcutaneous injection of D-gal (100 mg/kg) for 60 consecutive days, whereas the control group received an equal volume of PBS. Starting on day 31, the fisetin-treated groups were administered fisetin via daily oral gavage, while both the model and control groups received an equal volume of the blank vehicle mixture on the same schedule. During the experiment, the body weight of the mice was recorded every 6 days until the end of the experiment to assess weight changes. At 24 h after the last drug administration, ten mice in each group were weighed and euthanized. Whole blood, uteri, and ovaries were immediately harvested, weighed, and photographed. Five ovaries from each group were randomly collected and fixed in 4% paraformaldehyde (PFA) for morphological examination, and the remaining ovaries were rapidly frozen in liquid nitrogen and subsequently transferred to a −80 °C freezer for further biochemical analysis, Western blot or quantitative real-time polymerase chain reaction (qRT-PCR) analysis. In addition, to evaluate the ovarian GCs proliferation, the 5-Bromo-2′-deoxyuridine (BrdU, ST1056, Beyotime, Hangzhou, China) was dissolved in PBS at 10 mg/mL and then intraperitoneally injected at a volume of 200 μL per mouse into three mice per group. Both ovaries were harvested 4 h after BrdU injection and processed for fixation with 4% PFA.

### 2.2. Estrous Cycles and Hormone Assay

After 30 days of D-gal intervention, vaginal smears were collected from all mice every 6 days from 08:00 to 09:00 using the saline douching method until the end of the administration period. Vaginal lavage samples were smeared onto clear glass slides, air-dried, fixed in 70% ethanol for 10 min, and then stained with Giemsa (G1010, Solarbio, Beijing, China) for 20 min. After rinsing with distilled water and dried, the slides were observed and imaged using an Eclipse 80i microscope (Nikon, Tokyo, Japan). The normal estrous cycle consists of four stages: proestrus, characterized predominantly by irregularly shaped nucleated epithelial cells with few leukocytes or keratinized epithelial cells; estrus, composed almost entirely of lamellar anucleated keratinized cells; metestrus, in which a small number of non-nucleated keratinized epithelial cells appear alongside a leukocyte-predominant population; and diestrus, marked by crinkled epithelial cells and a large number of leukocytes. For hormone analysis, the blood samples were clotted at room temperature for 2 h, and then centrifuged at 3000 rpm at 4 °C for 15 min to obtain the serum. Serum concentrations of estradiol (E_2_) and progesterone were measured using commercial ELISA kits. The Mouse E_2_ ELISA Kit (JHN80666, Jinhengnuo, Hangzhou, China) has a detection range of 0.31–20 ng/mL and a sensitivity of less than 0.06 ng/mL, while the Mouse Progesterone ELISA Kit (JHN80652, Jinhengnuo) has a detection range of 1.56–100 ng/mL and a sensitivity of less than 0.28 ng/mL. All kits exhibit intra-assay coefficients of variation (CV) less than 9% and inter-assay CV less than 10%, with no cross-reactivities related structural analogs. All procedures were performed in strict accordance with the standard protocols of kits, with each sample and standard measured in duplicate. Absorbance was read at 450 nm using a microplate reader (Bio-Rad, Hercules, CA, USA).

### 2.3. Examination of Female Fertility

Following 60 days of drug administration, five females per group were selected to mate with young male BALB/c mice to evaluate female fertility. Briefly, these females were housed in harem breeding at a 2:1 female-to-male ratio for either 5 or 10 days, depending on the group, after which gestation was allowed to proceed for another two weeks. Day 1 of pregnancy was confirmed by the presence of a copulatory plug. To avoid inaccuracies in litter size due to cannibalism, females were euthanized before delivery, and the number of embryos was counted in utero.

### 2.4. Morphological Observation and Follicle Counts

The PFA-fixed ovarian tissues were rinsed under the running water overnight, dehydrated through a graded ethanol series, embedded in paraffin, and serially sectioned at a thickness of 5 μm. Every fifth section was mounted on a clear glass slide and stained with hematoxylin and eosin (H&E) following the standard laboratory protocols. The primordial, primary, secondary, and atretic follicles were identified and counted under an Eclipse 80i microscope (Nikon). In brief, follicles were classified as follows: primordial follicles, identified by an oocyte surrounded by a single layer of flattened GCs; primary follicles, containing an oocyte enclosed by a single layer of cuboidal GCs; secondary follicles, characterized by multiple layers of GCs with initial formation of small antral spaces but no fully formed antrum; and atretic follicles, distinguished by irregularly shaped oocytes and disordered arrangement of GCs. The number of follicles in each group was counted three times by different experimenters. To evaluate collagen deposition, ovarian sections from each group were subjected to Masson’s trichrome staining (G1340, Solarbio).

### 2.5. RNA Sequencing (RNA-Seq) Analysis

Total RNA was extracted from ovarian tissues of the control, D-gal, D-gal+fisetin (10 mg/kg) groups with a Rapid RNA Extraction Kit (400-100, Gooniebio, Guangzhou, China) according to the manufacturer’s instructions. Three independent biological replicates were used for each experimental condition. The total RNA samples were sent to Novogene (Beijing, China) for quality control, cDNA libraries construction, and sequencing, followed by transcriptome data analysis. Analysis of differentially expressed genes (DEGs) was performed using the DESeq2 R package (v1.38.0) with a threshold of |log_2_(FoldChange)| ≥ 0.5 and a *p*-value < 0.05. Kyoto Encyclopedia of Genes and Genomes (KEGG) enrichment analyses of the identified DEGs were performed using the R package *clusterProfiler* (v4.6.0).

### 2.6. GCs Culture and Treatments

The female BALB/c mice (4 weeks old) were prepared and intraperitoneally injected with 5 IU PMSG. After 48 h, mice were euthanized, both ovaries were rapidly harvested and rinsed three times with ice-cold PBS. Follicular fluid containing GCs and oocytes was collected by puncturing follicles with a 1 mL syringe needle, then filtered through a 200-mesh steel sieve (75 μm), and centrifuged at 1500 rpm for 5 min. After two washes with PBS, the GCs were resuspended and seeded onto 6-well plates using DMEM/F12 medium (Hyclone, Tauranga, New Zealand) supplemented with 10% fetal bovine serum (FBS, Hyclone, Logan, UT, USA) and 1% penicillin/streptomycin. The cells were placed in a 5% CO_2_ incubator at 37 °C for cell attachment. The GCs purity was identified by follicle stimulating hormone receptor (FSHR) immunofluorescence staining. GC purity was calculated as the percentage of FSHR-positive cells, and only samples with ≥90% purity were used for subsequent experiments. The identified GCs were seeded into a 96-well plate and cultured with the DMEM/F12 medium containing different concentrations of fisetin (0, 2.5, 5, 10, 20, 40, 80 µM) for 36 h to evaluate the cytotoxicity of fisetin. To examine the effect of fisetin on D-gal-induced senescence in GCs, cells were pretreated with fisetin for 36 h and then exposed to 200 mM D-gal for an additional 24 h. The concentration of D-gal used was based on our previous study [3]. For inhibitor experiments, GCs were pretreated with Compound C, an AMPK inhibitor, for 2 h prior to fisetin exposure. Compound C (HY-13418A, MedChemExpress, Shanghai, China) was dissolved in DMSO to a concentration of 100 mM and diluted to 10 μM for use in this experiment. To examine autophagic flux, GCs were treated with fisetin and D-gal, followed by 3-Methyladenine (3-MA, HY-19312, MedChemExpress) for another 6 h.

### 2.7. Cell Viability Assay

Cell viability was assessed using a Cell Counting Kit-8 (CCK-8, C0037, Beyotime) following the manufacturer’s instructions. Briefly, GCs were seeded in 96-well plates at 100 μL per well, allowed to adhere for 4 h, and then subjected to experimental treatments. Afterward, 10 µL of CCK-8 reagent was added to each well and incubated for an additional 2 h at 37 °C. Absorbance was measured at 450 nm using a microplate reader (Bio-Rad).

### 2.8. Immunofluorescence (IF)

The ovarian sections were deparaffinized, rehydrated, subjected to antigen retrieval in boiling 10 mM sodium citrate buffer (pH 6.0) for 20 min, and then blocked with 10% normal goat serum for 20 min at room temperature. GCs were seeded into the glass bottom confocal culture dish (FCFC020, Beyotime) and treated as mentioned above. Afterward, the GCs were fixed in 4% PFA for 15 min, rinsed three times with PBS, permeabilized with 0.5% Triton X-100 for 10 min, and blocked with 10% normal goat serum for 20 min. The ovarian sections and GCs were incubated overnight at 4 °C with anti-BrdU (1:200, G3G4; DSHB, Iowa City, IA, USA), anti-LC3 (1:200, ET1701-65, Huabio, Hangzhou, China), or anti-TOMM20 (1:100, ET1609-25, Huabio). After three washes in PBS, samples were incubated with the Alexa Fluor 594-conjugated Goat Anti-Rabbit IgG (1:500, AS039, ABclonal, Wuhan, China) or Alexa Fluor 488-conjugated Goat Anti-Rabbit IgG (1:200, AS053, ABclonal) for 1 h at 37 °C in the dark. The TdT-mediated dUTP nick end labeling (TUNEL) assay (A111-01, Vazyme, Nanjing, China) was performed to evaluate GCs apoptosis following the manufacturer’s protocol. Nuclei were further stained with DAPI (P0131, Beyotime) for 5 min at room temperature. Images were captured using laser confocal microscopy (Olympus IX81-FV1000, Tokyo, Japan) and analyzed by ImageJ v2.3.0 software (NIH, Bethesda, MD, USA). For BrdU analysis, at least 5 random microscopic fields containing different stages of follicles were captured from three mice per group. The total number of BrdU-positive nuclei and DAPI-stained nuclei was counted per field using ImageJ v2.3.0 software (NIH). The percentage of BrdU-positive cells was calculated as the ratio of BrdU-positive nuclei to DAPI-stained nuclei.

### 2.9. Western Blot (WB) Analysis

Total protein from ovarian tissues and GCs was extracted using ice-cold RIPA buffer (P0013B, Beyotime) supplemented with phenylmethylsulphonyl fluoride (PMSF, ST506, Beyotime). A BCA protein assay kit (P0009, Beyotime) was used to detect the protein concentration. Protein samples were diluted to 2 µg/µL with loading buffer and denatured at 100 °C for 10 min. 10 µL protein samples were load onto a 10% sodium dodecyl sulfate-polyacrylamide gel electrophoresis (SDS-PAGE, 8.6 cm × 6.8 cm × 1.0 mm), separated at a constant voltage of 200 V for 1 h, and then electrotransferred onto nitrocellulose membranes (Millipore, Darmstadt, Germany) at a constant current of 200 mA for 45 min. The membranes were blocked with 5% non-fat milk dissolved in Tris-buffered saline Tween-20 (TBST, PH 7.4) for 1 h at room temperature, and then incubated overnight at 4 °C with the following primary antibodies: Bax (1:500, WL01637, Wanleibio, Shenyang, China), Bcl-2 (1:500, WL01556, Wanleibio), Caspase-3 (1:500, ER1802-42, Huabio), cyclin-dependent kinase 2 (CDK2, 1:500, R1309-3, Huabio), proliferating cell nuclear antigen (PCNA, 1:500, WL03213, Wanleibio), AMPK (1:500, WL02254, Wanleibio), p-AMPK (1:500, WL05103, Wanleibio), mTOR (1:500, WL02477, Wanleibio), p-mTOR (1:500, WL03694, Wanleibio), Parkin (1:1000, RT1702-60, Huabio), PINK1 (1:1000, HA723021, Huabio), LC3 (1:1000, ET1701-65, Huabio), translocase of outer mitochondrial membrane 20 (TOMM20, 1:1000, ET1609-25, Huabio), cyclin-dependent kinase 6 (CDK6, 1:1000, ER40101, Huabio), and β-actin (1:5000, EM2001-07, Huabio). Following three washes with TBST, the membranes were incubated with a secondary antibody for 1 h at room temperature. Immunoreactive bands were detected with a clarity ECL Western blot substrate kit (Bio-Rad, #1705061, Hercules, CA, USA), imaged on a ChemiScope 3400 Mini machine (Clinx, Shanghai, China), and quantified by ImageJ v2.3.0 software (NIH). Target protein expression was normalized to β-actin.

### 2.10. Measurement of CAT, T-SOD, MDA, and GSH

Ovarian tissues were homogenized in cold PBS and then centrifuged at 2500 rpm for 10 min at 4 °C to obtain a 10% tissue suspension, while GCs were ultrasonically disrupted to release intracellular components. Total protein concentration, CAT (A007-1-1), T-SOD (A001-1-1), MDA (A003-1-2), and GSH (A006-2-1) levels were measured using corresponding assay kits (Nanjing Jiancheng Bioengineering Institute, Nanjing, China), following the manufacturer’s instructions.

### 2.11. SA-β-Gal Staining

A β-galactosidase staining kit (G1580, Solarbio) was used to detect the senescence-specific β-galactosidase expression in treated GCs according to the kit’s protocols. Briefly, the GCs were fixed in β-galactosidase fixative solution for 15 min, rinsed three times with PBS, and then incubated with 1 mL dyeing liquid (10 µL β-galactosidase staining fluid A, 10 µL fluid B, 930 µL fluid C, and 50 µL X-Gal solution) overnight at 37 °C. Images were captured with an Eclipse 80i microscope (Nikon) and quantified using ImageJ v2.3.0 software (NIH) to distinguish blue cells (positive) from unstained cells (negative) by setting a color threshold.

### 2.12. Cell-Cycle Distribution

After treatments, GCs were collected by trypsinization, washed twice with PBS, and fixed with ice-cold 75% ethanol, followed by resuspension in 500 µL of propidium iodide solution (480 µL staining buffer, 10 µL PI, and 10 µL RNase A) and incubation for 30 min. Cell cycle distribution was performed using a FACSCalibur flow cytometer (BD Biosciences, Franklin Lakes, NJ, USA), and analyzed by FlowJo v10.8.1 software (FlowJo, LLC, Ashland, OR, USA).

### 2.13. RNA Extraction and qRT-PCR

Total RNA was extracted from ovarian tissues and GCs using TRIzol reagent (15596018, Life Technologies, New York, NY, USA) in accordance with standard protocols, followed by assessment of its integrity and purity using a microspectrophotometer. The HiScript II 1st Strand cDNA Synthesis Kit (R211-01, Vazyme) was used to reverse-transcribe 1 µg RNA into cDNA, which was then amplified by qRT-PCR using the HiScript II One Step qRT-PCR SYBR Green Kit (Q221-01, Vazyme) to evaluate gene expression. The primers are listed in Table S1. Relative mRNA levels were calculated by the 2^−∆∆Ct^ method and normalized to the internal control β-actin.

### 2.14. Determination of Mitochondrial Membrane Potential (MMP) and ROS

The MMP of GCs was evaluated using an enhanced mitochondrial membrane potential assay kit with the fluorescent probe JC-1 (C2003S, Beyotime) following the instructions. Briefly, the treated GCs were rinsed twice with PBS, incubated with 1 mL of JC-1 working solution for 20 min at 37 °C in a 5% CO_2_ incubator with saturated humidity, and finally, the cells were rinsed twice with premade JC-1 staining buffer and examined with a confocal laser scanning microscope (Olympus IX81-FV1000). The red/green fluorescence intensity ratio was calculated by ImageJ v2.3.0 software (NIH) to analyze mitochondrial depolarization. For intracellular ROS assessment, the treated GCs were incubated with 10 µM fluorescent probe 2′,7′-dichlorofluorescein diacetate (DCFH-DA, S0033S, Beyotime) for 20 min at 37 °C, washed twice with ice-cooled PBS, and stained with Hoechst 33342 (C1027, Beyotime) for 5 min for nuclear visualization. The green fluorescence was examined under a confocal laser scanning microscope (Olympus IX81-FV1000) and measured using ImageJ v2.3.0 software (NIH). All experiments were performed in triplicate.

### 2.15. The 5-Ethynyl-2′-deoxyuridine (EdU) Assay

Cell proliferation was measured using an EdU assay kit (C0071S, Beyotime) following the manufacturer’s protocol. After incubation with 10 µM EdU for 2 h, the GCs were fixed in 4% PFA, washed twice with PBS and permeabilized with 0.5% Triton X-100 for 15 min at room temperature. Afterward, the specific GCs were washed with PBS three times and incubated with 0.5 mL click reaction solution (430 µL click reaction buffer, 20 µL CuSO_4_, 1 µL Azide 594, and 50 µL click additive solution) for 30 min in the dark. DAPI was used to mark the nucleic acids. Images were captured by a fluorescence microscope (Olympus IX70, Tokyo, Japan), and the EdU-positive cells were calculated by ImageJ v2.3.0 software (NIH).

### 2.16. Molecular Docking

The three-dimensional structure of fisetin was obtained from the PubChem database (https://pubchem.ncbi.nlm.nih.gov/), and the AMPK protein structure (PDB ID: 5UFU) was obtained from the Protein Data Bank (PDB, http://www.rcsb.org/) with the species set to Mus musculus (house mouse). The AMPK receptor was prepared by removing water molecules and adding hydrogen atoms, after which its potential binding sites were predicted. The ligand, fisetin, was subjected to conformational search and energy minimization. Molecular docking was performed using AutoDock Vina (v1.2.0), and three-dimensional images of the docking results were generated using the PyMOL Molecular Graphics System (v2.5.0). 

### 2.17. Transmission Electron Microscopy (TEM)

The collected GCs were fixed overnight in 2.5% glutaraldehyde at 4 °C, postfixed in buffered 1% osmium tetroxide for 1.5 h, dehydrated through a graded ethanol or acetone series, and embedded in propylene oxide resin following standard TEM procedures. Ultrathin sections (70–90 nm) were cut on an ultramicrotome (Leica EM UC7, Wetzlar, Germany), stained with 8% aqueous uranyl acetate and Reynold’s lead citrate, and examined under a Tecnai G2 Spirit (FEI Company, Hillsboro, OR, USA) at 120 kV at various magnifications.

### 2.18. Statistical Analysis

Data are expressed as mean ± SEM. One-way analysis of variance (ANOVA) followed by Tukey’s or Dunnett’s test was employed to assess differences between groups, with *p* < 0.05 considered statistically significant.

## 3. Results

### 3.1. Fisetin Alleviates D-Gal-Induced Impairment of Ovarian Function and Fertility in Mice

**Figure 1:**
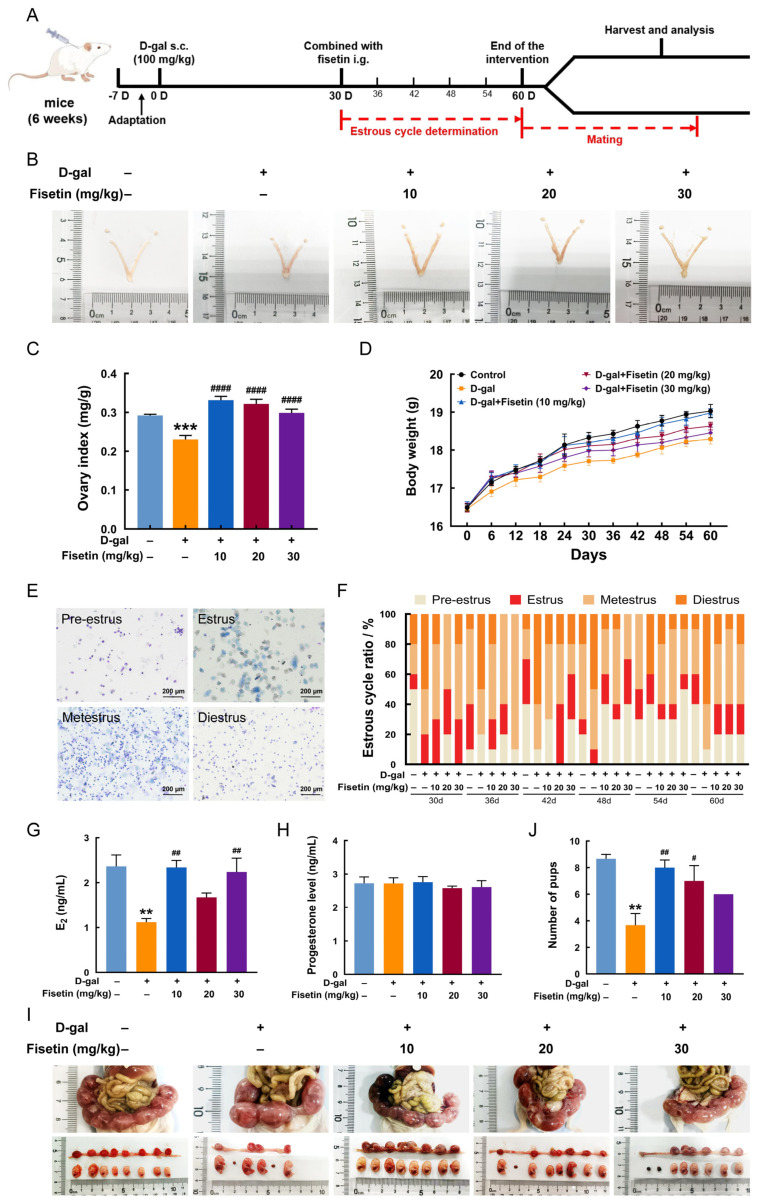
Fisetin regularized the estrus cycle and improved reproductivity in D-gal-induced aging mice. (**A**) Diagram of experimental design. (**B**) Representative images of uterine and ovaries morphology in mice across groups. (**C**) Ovarian index in mice (n = 10). (**D**) Body weight changes in mice during experimental period (n = 10). (**E**) Representative images of mouse vaginal smears. Scale bar: 200 μm. (**F**) Percentage of pre-estrus, estrus, metestrus, and diestrus in mice from each group. (**G**,**H**) Serum estrogen and progesterone levels in mice (n = 10). (**I**,**J**) Representative results of mating experiments and pup count in mice from each group (n = 3). ** *p* < 0.01, *** *p* < 0.001 vs. control group, ^#^ *p* < 0.05, ^##^ *p* < 0.01, ^####^ *p* < 0.0001 vs. D-gal group.

A D-gal-induced ovarian aging model was established to explore the effect of fisetin on ovarian injury (Figure 1A). As shown in Figure 1B,C, the ovarian index, calculated as the ovary weight-to-body weight ratio, was significantly decreased in the D-gal group, but was markedly recovered following fisetin supplementation at 10, 20 or 30 mg/kg. All mice survived the experimental period, exhibiting no significant differences in body weight among groups (Figure 1D). Estrous cycle disorder serves as a key indicator of endocrine dysfunction. Vaginal smears were collected periodically from day 30 to 60 of administration to monitor the estrous cycle. Results showed that estrous cycle disruption occurred in all D-gal model mice by day 30, in contrast to the control group, which maintained a normal cycle, confirming successful induction of ovarian endocrine dysfunction. This disruption was significantly ameliorated following 18 days of fisetin treatment (Figure 1E,F). In addition, the significant decrease in serum estradiol level observed in the D-gal-treated mice, compared with the control group, was reversed by fisetin treatment at 10 or 30 mg/kg (Figure 1G). However, there was no significant difference in serum progesterone level among groups (Figure 1H). Mating experiments were performed after treatment to further assess reproductive function, with embryo numbers quantified per group. Representative images were shown in Figure 1I. Compared with the control group, the D-gal group exhibited a significant decrease in the average number of pups, which was considerably reversed in groups co-treated with fisetin at 10 or 20 mg/kg (Figure 1J).

### 3.2. Fisetin Reduces D-Gal-Induced Follicular Atresia, Ovarian Fibrosis and Senescence in Mice

**Figure 2:**
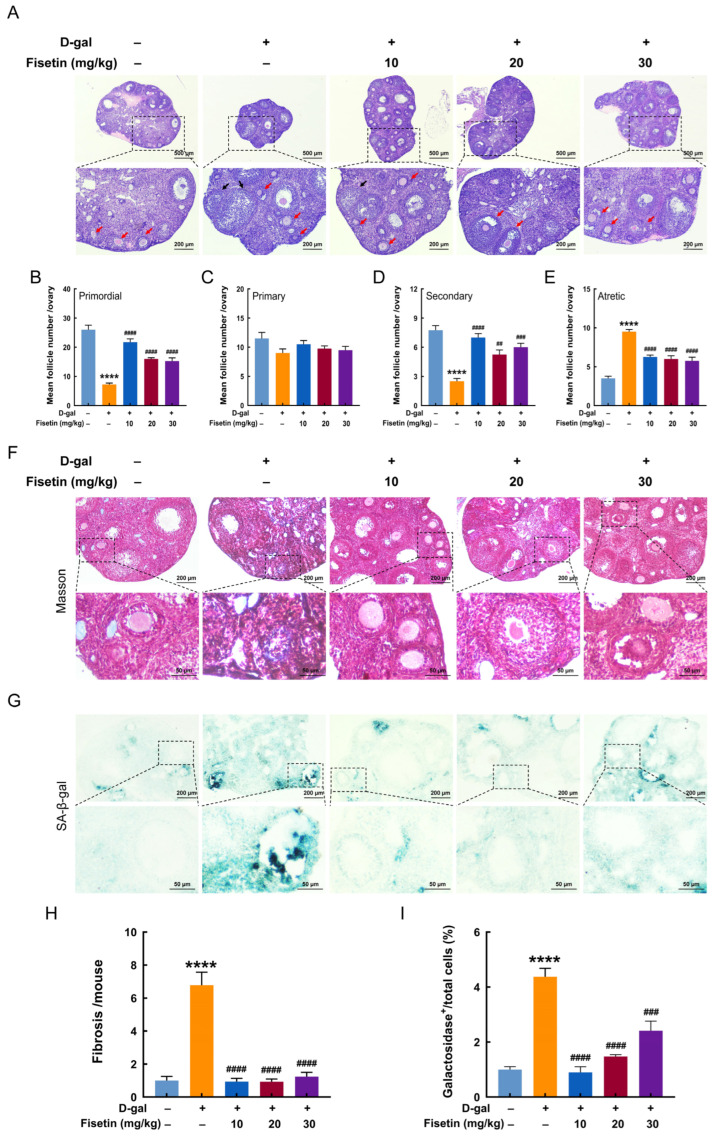
Fisetin improved ovarian follicular atresia, fibrosis and senescence in D-gal-induced aging mice. (**A**) Representative H&E staining of ovaries in each group. Red and black arrows indicate growing follicles and atretic follicles, respectively. Scale bars: 500 μm and 200 μm. (**B**–**E**) Counts of primordial, primary, secondary, and atretic follicles in each group (n = 5). (**F**) Masson’s trichrome staining of ovaries in mice from each group. Scale bars: 200 μm and 50 μm. (**G**) Representative images of SA-β-gal staining in ovarian sections from each group. Scale bars: 200 μm and 50 μm. (**H**,**I**) Quantification of (**F**) and (**G**), respectively (n = 5). **** *p* < 0.0001 vs. control group, ^##^ *p* < 0.01, ^###^ *p* < 0.001, ^####^ *p* < 0.0001 vs. D-gal group.

Representative images of H&E-stained ovaries were shown in Figure 2A. Compared with the control group, the D-gal group showed more atretic follicles, while fisetin supplementation increased the number of growing follicles. Follicular count indicated that the D-gal group exhibited a significant reduction in the number of primordial and secondary follicles, but an increase in atretic follicles, relative to the control group. However, this phenomenon was reversed by fisetin supplementation at 10, 20 or 30 mg/kg (Figure 2B,D,E). No significant difference was found in the number of primary follicles among groups (Figure 2C). Masson’s trichrome staining revealed that darker blue color, indicative of collagen fibers, was observed in the ovarian follicular stroma of the D-gal group, while only weak staining was found in the other groups. Quantitative analysis showed that the content of collagen fibers in ovaries of the D-gal group was significantly higher than that in the control group, while it was lower than that in the fisetin-treated groups (Figure 2F,H). Similarly, SA-β-gal staining demonstrated a significantly increased proportion of senescence-positive cells in ovarian tissues of the D-gal group, while fisetin treatment markedly reduced this proportion, with the level in the 10 mg/kg fisetin group comparable to that in the control group (Figure 2G,I).

### 3.3. Fisetin Inhibits D-Gal-Induced Ovarian Oxidative Stress and GC Apoptosis in Mice

**Figure 3:**
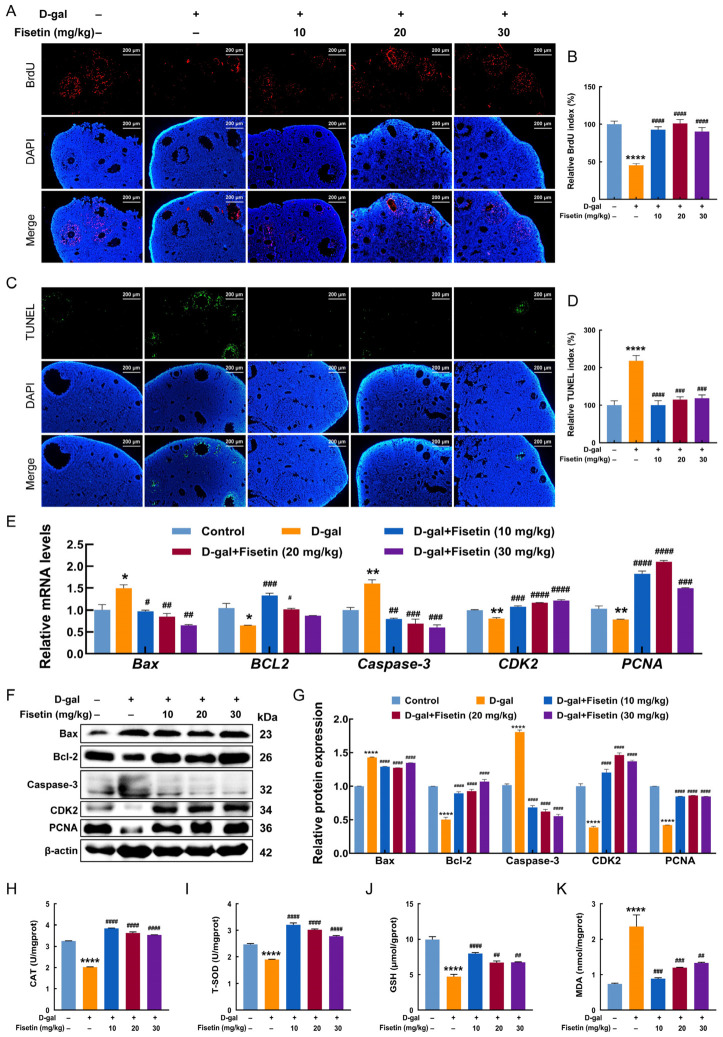
Fisetin prevented ovarian oxidative stress and GC apoptosis but promoted proliferation in D-gal-induced aging mice. (**A**–**D**) BrdU incorporation and TUNEL labeling of ovaries in mice from each group. Scale bar: 200 μm. (**E**) qRT-PCR for *Bax*, *BCL-2*, *Caspase-3*, *CDK2*, and *PCNA* mRNA levels of ovaries in mice from each group. (**F**,**G**) Protein levels of Bax, Bcl-2, Caspase-3, CDK2, and PCNA of ovaries in mice from each group. (**H**–**K**) Measurement of ovarian T-SOD and CAT activities, as well as GSH and MDA levels, in mice from each group. * *p* < 0.05, ** *p* < 0.01, **** *p* < 0.0001 vs. control group, ^#^ *p* < 0.05, ^##^ *p* < 0.01, ^###^ *p* < 0.001, ^####^ *p* < 0.0001 vs. D-gal group.

As shown in Figure 3A,B, BrdU incorporation assays indicated that D-gal treatment significantly reduced follicular GC proliferation compared with the control group. However, supplementation with fisetin at 10, 20 or 30 mg/kg markedly increased the percentage of BrdU-positive GCs and effectively restored GC proliferative capacity. TUNEL results showed a significantly increased percentage of TUNEL-positive GCs in the D-gal group compared with the control group, which was decreased after fisetin intervention (Figure 3C,D). Consistent with these findings, qRT-PCR and Western blotting revealed that D-gal significantly downregulated the expression of the proliferation-related markers PCNA and CDK2 and the anti-apoptotic marker Bcl-2, while upregulating the apoptotic markers Bax and Caspase-3 at both mRNA and protein levels (Figure 3E–G). Furthermore, D-gal treatment induced the significant reductions in ovarian antioxidant enzyme activities, including T-SOD and CAT, as well as the level of GSH, while causing an increase in MDA production. However, fisetin supplementation at 10, 20 or 30 mg/kg prevented these changes effectively (Figure 3H–K).

### 3.4. Transcriptomic Analysis of the Effects of Fisetin on D-Gal-Induced Ovarian Damage in Mice

**Figure 4:**
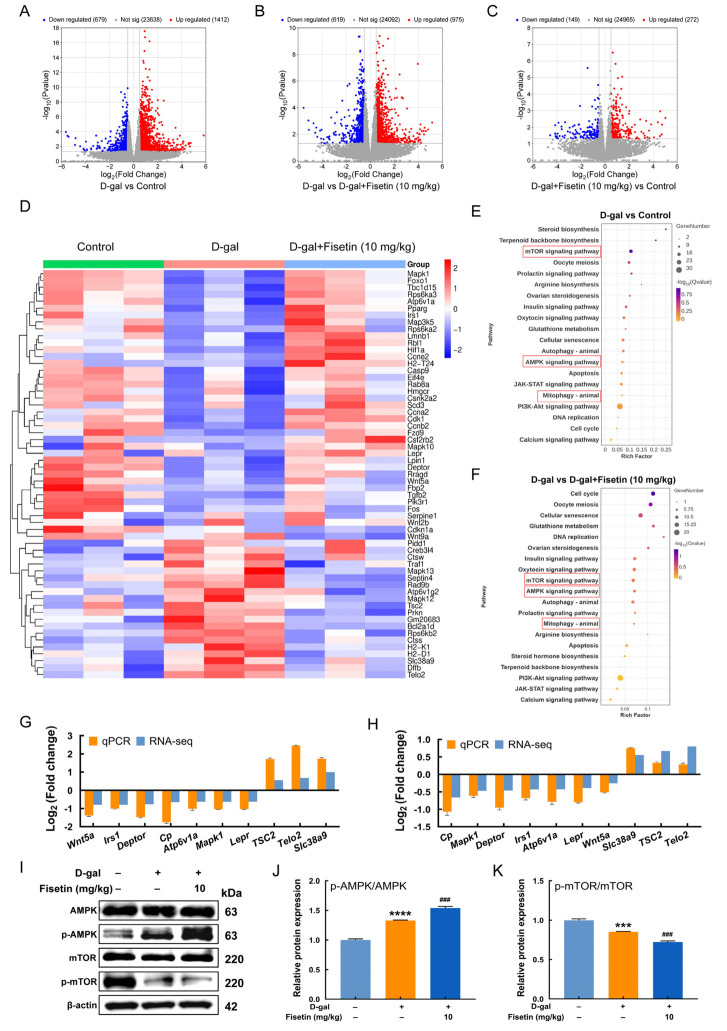
Effect of fisetin on ovarian gene expression profiles in D-gal-induced aging mice. (**A**–**C**) Volcano plot of DEGs distribution. (**D**) Cluster heatmap of DEGs. Columns and rows represent samples and genes, respectively. Red and blue indicate up-and down-regulation, respectively. (**E**,**F**) KEGG enrichment analysis of DEGs in D-gal group vs. control group (**E**), and D-gal+fisetin group vs. D-gal group (**F**). (**G**,**H**) Comparison of RNA-seq data and qRT-PCR validation of 10 DEGs between the D-gal group vs. control group (**G**), and between the D-gal group and D-gal+fisetin group (**H**). (**I**–**K**) Western blot and quantitative analysis of p-AMPK/AMPK and p-mTOR/mTOR proteins in ovaries from control, D-gal, and D-gal+fisetin groups. *** *p* < 0.001, **** *p* < 0.0001 vs. control group, ^###^ *p* < 0.001 vs. D-gal group.

To further investigate the effect of fisetin on D-gal-induced ovarian damage, ovarian tissues from the control, D-gal, and D-gal+fisetin (10 mg/kg) groups were collected for RNA-seq analysis. DEGs were visualized using volcano plots for each comparison (Figure 4A–C). The results revealed that compared with the control group, D-gal induced the upregulation of 1412 genes and downregulation of 679 genes, while the D-gal group supplemented with fisetin induced 272 upregulated and 149 downregulated genes. In the comparison between the D-gal and the fisetin-supplemented D-gal groups, 975 genes were upregulated while 619 were downregulated, further confirming the protective effect of fisetin. A clustered heatmap demonstrates distinct expression patterns of DEGs among groups (Figure 4D). Apart from cell senescence- and apoptosis-related gene abnormalities, differences were also observed in mRNAs associated with mitophagy and AMPK/mTOR signaling pathway. Compared with the control group, D-gal treatment altered the expression of mitophagy-related DEGs (e.g., *TBC1 domain family member 15* (*Tbc1d15*), *hypoxia inducible factor 1 subunit alpha* (*Hif1a*), *casein kinase 2 alpha 2* (*Csnk2a2*), *mitogen-activated protein kinase 10* (*Mapk10*), *Gm20683*) and also affected AMPK/mTOR-related DEGs (e.g., *TSC complex subunit 2* (*Tsc2*), *Ras-related protein Rab-8A* (*Rab8a*), *fructose-bisphosphatase 2* (*Fbp2*), *mitogen-activated protein kinase 1* (*Mapk1*), *ribosomal protein S6 kinase B2* (*Rps6kb2*)). Furthermore, relative to the D-gal group, fisetin supplementation in D-gal-treated mice altered additional mitophagy-related DEGs (e.g., *Tbc1d15*, *Hif1a*, *Mapk10*, *Prkn*, *Gm20683*) and AMPK/mTOR-related DEGs (e.g., *peroxisome proliferator activated receptor gamma* (*Pparg*), *stearoyl-coenzyme A desaturase 3* (*Scd3*), *cyclin A2* (*Ccna2*), *insulin receptor substrate 1* (*Irs1*), *wnt family member 9A* (*Wnt9a*), *cytosolic arginine sensor for mTORC1 subunit 1* (*Castor1*)). KEGG enrichment results indicated that the DEGs between the D-gal group and control group were primarily enriched in mTOR signaling pathway, cellular senescence, AMPK signaling pathway, mitophagy, and the PI3K-Akt signaling pathway (Figure 4E). For the comparison between the D-gal group and fisetin-supplemented D-gal group, the DEGs were predominantly enriched in mTOR signaling pathway, AMPK signaling pathway, mitophagy, and apoptosis (Figure 4F). This bioinformatic analysis confirmed that AMPK/mTOR signaling pathway served as a key factor in fisetin-mediated alleviation of D-gal-induced ovarian injuries and had key associations with cellular senescence, apoptosis, and mitophagy. Ten DEGs, including *wnt family member 5A* (*Wnt5a*), *Irs1*, *DEP domain containing mTOR interacting protein* (*Deptor*), *ceruloplasmin* (*Cp*), *Mapk1*, *ATPase H^+^ transporting V1 subunit A* (*Atp6v1a*), *leptin receptor* (*Lepr*), *Tsc2*, *telomere maintenance 2* (*Telo2*), and *solute carrier family 38 member 9* (*Slc38a9*), were randomly selected for qRT-PCR verification using ovarian samples from each group. As shown in Figure 4G,H, the expression patterns of all genes were highly consistent with the RNA-seq results, confirming the reliability of the RNA-seq data and the correctness of transcript identification. In addition, Western blotting results revealed that, compared with the D-gal group, the D-gal+fisetin group exhibited a significantly higher p-AMPK/AMPK ratio but a lower p-mTOR/mTOR ratio, indicating AMPK activation and mTOR inhibition (Figure 4I–K).

### 3.5. Fisetin Induces PINK1/Parkin-Mediated Mitophagy in D-Gal-Treated Ovaries and GCs

**Figure 5:**
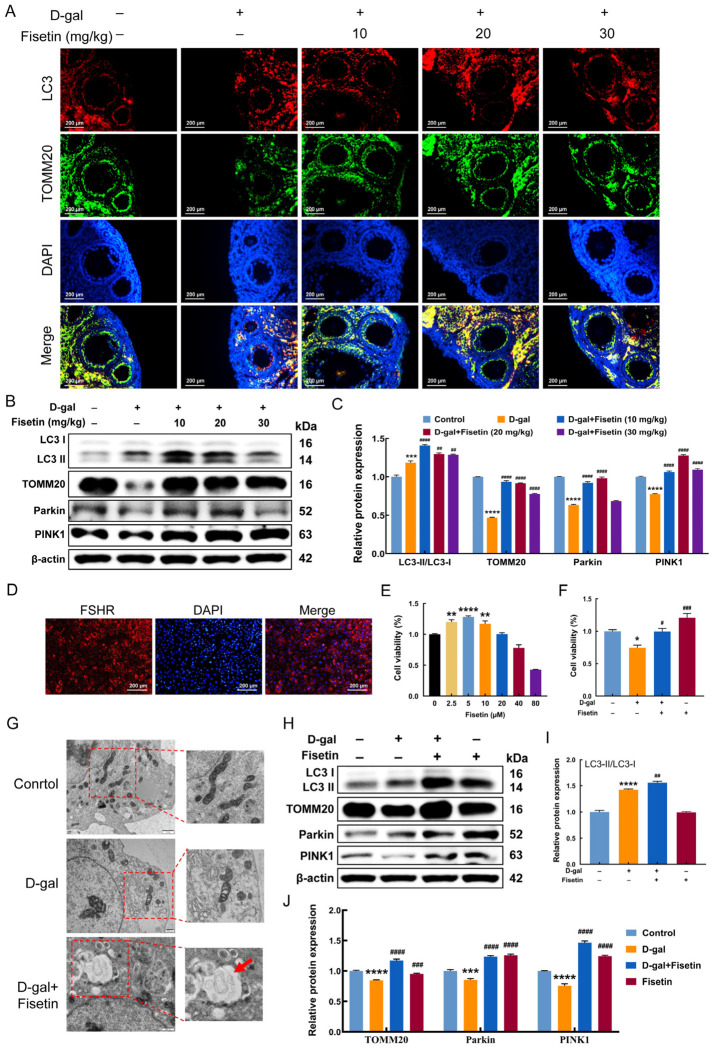
Fisetin induced mitophagy in D-gal-treated ovaries and GCs through the PINK1-Parkin signaling pathway. (**A**) Representative immunofluorescence images of LC3 and TOMM20 co-staining of ovaries in mice from each group. Scale bar: 200 μm. (**B**,**C**) Western blot analysis of LC3-II/I, TOMM20, PINK1, and Parkin in treated mouse ovarian tissues from each group. (**D**) FSHR immunofluorescence identification of mouse GCs. Scale bar: 200 μm. (**E**) Effect of different concentrations of fisetin on GC viability as measured by CCK-8 (n = 6). (**F**) Cell viability of GCs exposed to fisetin and 200 mM D-gal was measured using the CCK-8 assay. (n = 6). (**G**) TEM imaging of mitochondria and mitophagosomes (red arrow) in GCs following D-gal and fisetin treatment. Scale bars: 1 μm and 500 nm. (**H**–**J**) Protein expression and quantification of LC3-II/I, TOMM20, Parkin, and PINK1 were determined in GCs exposed to D-gal and fisetin through Western blotting. * *p* < 0.05, ** *p* < 0.01, *** *p* < 0.001, **** *p* < 0.0001 vs. control group, ^#^ *p* < 0.05, ^##^ *p* < 0.01, ^###^ *p* < 0.001, ^####^ *p* < 0.0001 vs. D-gal group.

Immunofluorescence co-staining of TOMM20 and LC3 was performed to assess the interaction between mitochondria and the autophagosome in ovarian tissues, an indicator of mitophagy. The D-gal group showed significantly less co-localization of LC3 and TOMM20 than the control group, indicating impaired mitophagy, whereas administration of fisetin, particularly at 10 and 20 mg/kg, increased this co-localization, suggesting enhanced mitophagy (Figure 5A). Western blotting corroborated this finding. Fisetin treatment at 10 and 20 mg/kg induced a significant increase in LC3-II/I protein level and reversed the D-gal-induced decline in PINK1, Parkin, and TOMM20 expression (Figure 5B,C). FSHR immunofluorescence confirmed that the isolated mouse GCs had a purity exceeding 90%, meeting the requirements for subsequent experiments (Figure 5D). Examination of cell viability after 36 h exposure to different concentrations of fisetin revealed that 2.5, 5, and 10 μM significantly increased GC viability compared to the control, with 5 μM being the most effective (Figure 5E). As expected, 5 μM fisetin effectively counteracted the D-gal-induced decline in GC viability (Figure 5F). The results of TEM revealed dramatically shrunken mitochondria in D-gal-induced GCs, while in fisetin-treated GCs, mitophagosomes (red arrow) were observed as double-membrane vesicular structures that enveloped the injured mitochondria (Figure 5G). Consistently, WB assay demonstrated significant LC3-II accumulation and markedly elevated expression levels of PINK1, Parkin, and TOMM20 in fisetin-treated senescent GCs relative to the control and D-gal-treated groups (Figure 5H–J).

### 3.6. Fisetin Promotes Mitophagy via the Activation of AMPK/mTOR Signaling in GCs

**Figure 6:**
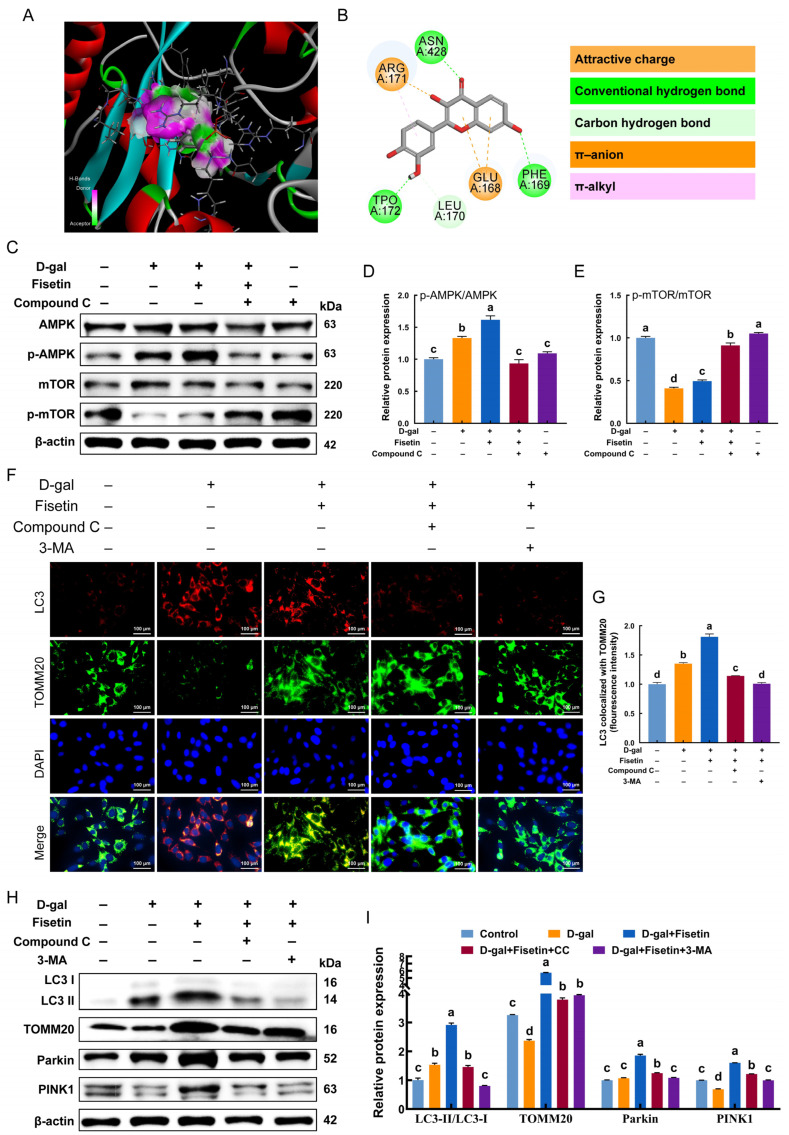
Involvement of AMPK in the activation of fisetin-mediated mitophagy in D-gal-induced senescent GCs. (**A**) 3D molecular representation of fisetin and AMPK. (**B**) 2D interaction diagram of fisetin with AMPK. (**C**–**E**) Western blot analysis of p-AMPK/AMPK and p-mTOR/mTOR expression in control, D-gal, D-gal+Fisetin, Compound C alone, and Compound C+D-gal+Fisetin groups. (**F**,**G**) Immunofluorescence co-staining of mitophagic markers LC3 and TOMM20 was examined in GCs from control, D-gal, D-gal+Fisetin, D-gal+Fisetin+Compound C, D-gal+Fisetin+3-MA groups. Scale bar: 100 μm. (**H**,**I**) The protein levels of the LC3-II/I, TOMM20, PINK1, and Parkin in GCs following treatments described in (**F**) were assessed by Western blotting. Different lowercase indicated significant differences (*p* < 0.05).

To predict the direct interaction between fisetin and AMPK, a molecular docking was conducted. As shown in Figure 6A,B, fisetin exhibited a strong binding affinity for AMPK (binding energy: −44.8348 kcal/mol), primarily through multiple types of interactions, including conventional hydrogen bonds, π-anion interactions, and attractive charge interactions, with key residues Glu-168, Phe-169, Leu-170, Arg-171, and Thr-172. Consistently, Western blot analysis demonstrated that fisetin increased the p-AMPK/AMPK ratio and decreased the p-mTOR/mTOR ratio in D-gal-treated senescent GCs, whereas the addition of the AMPK inhibitor dorsomorphin (Compound C) reversed these alterations (Figure 6C–E). AMPK serves as a critical regulator of mitophagy, and its involvement was explored in subsequent experiments. Fluorescence co-localization of LC3 with TOMM20 revealed that the AMPK inhibitor attenuated the fisetin-mediated increase in mitophagosome formation in D-gal-induced senescent GCs (Figure 6F,G). Consistent with this finding, AMPK inhibition completely reversed fisetin-induced mitophagic activity, as evidenced by the reduction in the elevated levels of LC3-II/I, TOMM20, PINK1, and Parkin (Figure 6H,I).

### 3.7. Fisetin Attenuates D-Gal-Induced Mitochondrial Injury and Oxidative Stress by AMPK-Mediated Mitophagy

**Figure 7:**
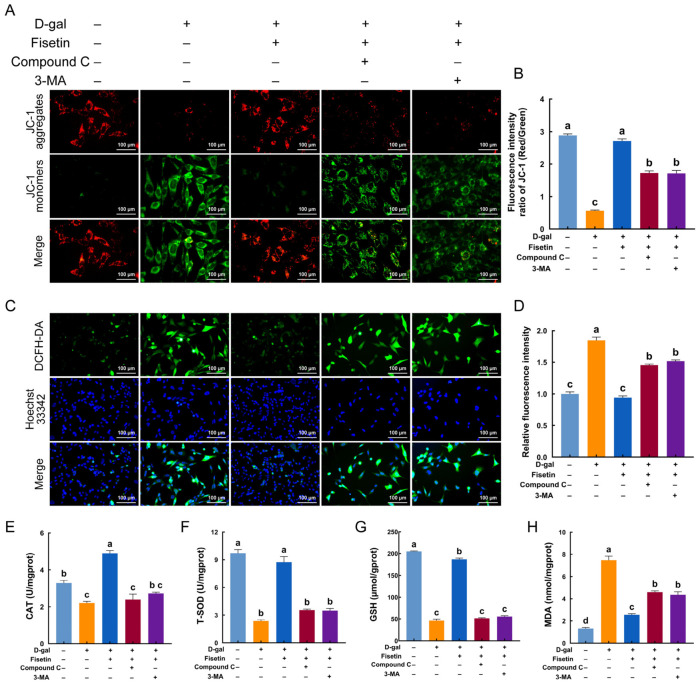
Fisetin protected against D-gal-induced mitochondrial injury and oxidative stress via AMPK-mediated mitophagy. (**A**,**B**) Representative images and quantification of mitochondrial membrane potential detected by JC-1 staining in control, D-gal, Fisetin+D-gal, Compound C+D-gal+Fisetin, and D-gal+Fisetin+3-MA groups. Scale bar: 100 μm. (**C**,**D**) Representative fluorescence images and quantification of ROS using DCFH-DA staining. Scale bar: 100 μm. (**E**–**H**) Levels of the antioxidants CAT, T-SOD, and GSH, as well as the oxidative product MDA, in GCs treated as described above. Different lowercase indicated significant differences (*p* < 0.05).

Mitophagy activation is closely associated with mitochondrial integrity and the production of intracellular ROS. The effects of fisetin-enhanced mitophagy on mitochondrial function were further assessed by detecting mitochondrial membrane potential and ROS levels. JC-1 staining revealed a significant increase in the aggregate-to-monomer ratio in fisetin-treated senescent GCs relative to D-gal alone, reaching levels comparable to the control. However, the addition of Compound C and an autophagy inhibitor, 3-MA, largely reversed this increase, demonstrating that fisetin’s ability to maintain mitochondrial integrity depends on AMPK-mediated mitophagy (Figure 7A,B). Mitochondrial dysfunction is always accompanied by pronounced oxidative stress. Fluorescence staining showed that fisetin-treated senescent GCs exhibited a significant reduction in ROS production compared to the D-gal group, but showed no significant difference from the control group, whereas treatment with Compound C and 3-MA significantly abrogated this change (Figure 7C,D). Similarly, inhibition of AMPK and autophagy abolished the fisetin-induced increase in the activities of antioxidant enzymes CAT and T-SOD as well as in GSH content in D-gal-induced senescent GCs (Figure 7E–G). In addition, the decrease in MDA level detected in fisetin-treated senescent GCs was reversed by inhibition of AMPK-mediated mitophagy (Figure 7H).

### 3.8. Mitophagy Inhibition Abolishes the Protective Effects of Fisetin Against D-Gal-Induced Cell Cycle Arrest and Senescence in GCs

**Figure 8:**
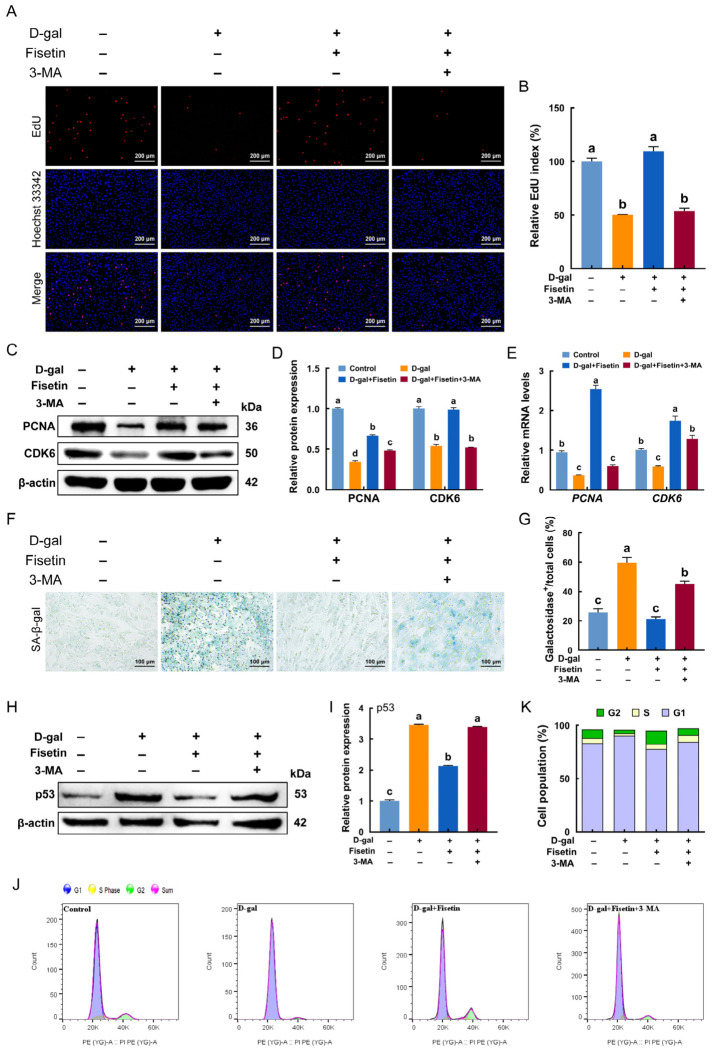
Fisetin alleviated D-gal-induced cell senescence and cell cycle arrest by activating mitophagy. (**A**,**B**) EdU assay in GCs following treatments of control, D-gal, Fisetin+D-gal, Fisetin+D-gal+3-MA. Scale bar: 200 μm. (**C**,**D**) Western blot analysis of PCNA and CDK6 in GCs following the treatments described above. (**E**) qRT-PCR analysis of *PCNA* and *CDK6* mRNA levels in the cultured GCs following treatments described in (**A**). (**F**,**G**) Percentage and representative micrographs of SA-β-gal-stained GCs relative to total cells. Scale bar: 100 μm. (**H**,**I**) The protein level of p53 was determined by Western blotting. (**J**,**K**) Flow cytometric analysis of cell-cycle distribution in treated GCs. Different lowercase indicated significant differences (*p* < 0.05).

Here, 3-MA, an autophagy inhibitor, was applied to suppress fisetin-induced mitophagy in senescent GCs, allowing subsequent detection of changes in cell proliferation and senescence. As shown in Figure 8A,B, EdU incorporation revealed that fisetin significantly increased the number of EdU-labeled cells in D-gal-induced senescent GCs, whereas this increase was attenuated by 3-MA addition. In accordance with this, 3-MA also significantly downregulated the expression of cell proliferation markers PCNA and CDK6 at both protein and mRNA levels (Figure 8C–E). β-galactosidase staining, a common marker of cellular senescence, further confirmed these findings. As presented in Figure 8F,G, pretreatment with fisetin markedly lowered the proportion of β-galactosidase-positive cells compared with the D-gal group, an effect reversed by 3-MA addition. Correspondingly, inhibition of mitophagy completely abolished the fisetin-mediated downregulation of p53 expression, suggesting that mitophagy contributes to suppressing cell senescence (Figure 8H,I). Furthermore, 3-MA treatment abolished the protective effect of fisetin against D-gal-induced cell cycle arrest (Figure 8J,K).

## 4. Discussion

The ovary is the organ most susceptible to aging, with a clear transition from a vigorous state to one characterized by shrinking volume, declining secretory function, and depletion of the non-growing follicle pool. During follicular development, GCs support oocyte maturation by synthesizing and transferring the necessary hormones and proteins, while these processes consume energy and generate substantial ROS [20]. ROS accumulation causes oxidative damage to GCs, resulting in mitochondrial dysfunction, DNA damage, and apoptosis, which in turn leads to follicular atresia and ovulation disorders [21]. It has been confirmed that dysfunction of GCs is linked to a variety of female reproductive disorders [22]. Therefore, timely supplementation of antioxidants is essential for preserving ovarian endocrine and reproductive function by delaying ovarian function degradation and reducing the oxidative damage to the ovary. In this study, we established a D-gal-induced aging model in mice and senescent GCs in vitro to explore how fisetin, a natural flavonoid, attenuates ovarian aging.

D-gal is a reducing monosaccharide routinely employed to establish senescence models in animals and cultured cells due to its simplicity, convenience, and cost-effectiveness. Chronic high-dose D-gal exposure in mice elicits a spectrum of fertility derangements, manifesting as disrupted estrous cycles, enhanced oxidative stress, and a significantly increased incidence of follicular atresia, all of which faithfully recapitulate the hallmarks of natural ovarian aging [23,24]. Fisetin, a natural bioactive flavonoid, has been recognized for its ability to treat a range of ovarian disorders due to its robust pharmacological properties, including antioxidant, anti-aging, and cytoprotective capacities. For instance, a previous study has shown that daily oral administration of fisetin for 14 consecutive days ameliorates hormonal disturbances, reverses metabolic imbalances, increases the expression of antioxidant genes, and suppresses the upregulated inflammatory response in the ovarian tissues of a mouse model of polycystic ovary syndrome [25]. Furthermore, in laying hens, fisetin supplementation has been evidenced to alleviate ovarian aging and follicular atresia by enhancing antioxidant capacity and glucose metabolism [26]. Consistently, our data revealed that fisetin supplementation significantly attenuated D-gal-induced reductions in ovarian index and serum E_2_ level, normalized estrous cycles, and enhanced fertility in mice. Ovarian reserve function, reflected by the primordial follicle pool size, serves as a key determinant of ovarian response and female fertility. In the present study, fisetin supplementation significantly attenuated D-gal-induced follicular atresia and increased the numbers of primordial and secondary follicles in mice. Furthermore, according to the body surface area (BSA) normalization method, the human equivalent dose (HED) can be calculated from the oral dose of 10 mg/kg/day used in mice of this study, yielding approximately 0.81 mg/kg, which falls well within the range of oral fisetin doses already validated in human clinical trials [27]. For instance, multiple clinical trials have administered oral fisetin at comparable or much higher doses with good tolerability, including a study (NCT03430037) in which older women aged over 70 years received 20 mg/kg/day orally for 2 days per month over two months to assess whether fisetin reduces insulin resistance, bone resorption, and frailty in older women with gait disturbance [28]. Additionally, research indicated that fifteen healthy volunteers received a single dose of 1000 mg unformulated fisetin or a hybrid-hydrogel formulation containing 192 mg fisetin, with no adverse effects, and the latter achieved a 27-fold increase in bioavailability [29]. Collectively, our findings indicate that fisetin effectively delays aging-related ovarian dysfunction in mice, providing a mechanistic basis for its potential role in the treatment of ovarian diseases in female.

Oxidative stress acts as a major driver of ovarian aging, as excessive ROS accumulation damages GCs and ultimately causes the ovary to lose its normal function. ROS overproduction impairs cellular antioxidant defenses, as evidenced by decreased activities of enzymes such as T-SOD, CAT, and GSH, and subsequently activates DNA damage response pathways, leading to cell cycle arrest, which in turn inhibits cell proliferation and promotes apoptosis [30]. Exogenous antioxidant supplementation can reduce oxidative stress in the aging ovary, thereby preserving its physiological function and supporting follicle growth. Recent evidence has indicated that fisetin supplementation plays a critical role in improving antioxidant markers, elevating T-SOD and CAT activities, raising GSH level, and suppressing MDA and ROS production, thereby alleviating oxidative damage and promoting cell proliferation in diverse models, including porcine early embryonic culture in vitro [31], heart tissue of bleomycin-induced pulmonary fibrosis [32], and a cellular model of high phosphate-induced vascular calcification [33]. Consistent with these findings, our data revealed that oral administration of fisetin significantly enhanced the activities of antioxidant enzymes T-SOD and CAT, upregulated GSH level, and suppressed MDA accumulation in ovarian tissues of D-gal-induced aging mice. Moreover, fisetin markedly inhibited follicular GC apoptosis, downregulated the expression of pro-apoptotic proteins Bax and Caspase-3, elevated the anti-apoptotic protein Bcl-2 level, and simultaneously increased the levels of proliferation-related proteins PCNA and CDK2 in D-gal-treated ovaries. Collectively, these results demonstrate that fisetin attenuates ovarian oxidative stress and follicular GC apoptosis while supporting GCs proliferation and follicular growth in D-gal-induced aging mice.

To further explore the molecular basis of fisetin-mediated protection against ovarian dysfunction, RNA-seq was conducted to link the detected phenotypes to their relevant signaling cascades. Differential expression analysis suggested that fisetin supplementation significantly modulated ovarian homeostasis, as evidenced by marked enrichment of genes associated with mitophagy, as well as AMPK and mTOR signaling pathways. Mitophagy, a critical quality control mechanism that selectively removes damaged mitochondria to maintain intracellular homeostasis under oxidative stress, has been confirmed to play a vital role in attenuating ovarian oxidative damage. PINK1 and Parkin are central regulators of mitophagy, wherein the accumulation of PINK1 recruits and activates Parkin via phosphorylation, thereby promoting LC3 binding and autophagosome formation; accordingly, evidence indicates that loss of PINK1 impairs mitophagy and accelerates ovarian aging in mice [34]. TOMM20, a core subunit of the outer mitochondrial membrane translocase complex, serves as both a common marker of mitochondrial integrity and a substrate for Parkin-mediated ubiquitination [35]. In this study, we observed that fisetin treatment significantly activated mitophagy in D-gal-induced aging ovaries and senescent GCs, as evidenced by enhanced co-localization of TOMM20 and LC3 in ovarian tissues, the presence of mitophagosomes in senescent GCs, and elevated levels of LC3-II/LC3-I, TOMM20, PINK1, and Parkin. Furthermore, the addition of fisetin reversed mitochondrial dysfunction, reduced ROS accumulation, alleviated cell cycle arrest, and attenuated cellular senescence in cultured GCs under D-gal stimulation. However, inhibition of mitophagy with 3-MA abolished these protective effects of fisetin against D-gal-induced damage in follicular GCs. These results clearly indicate that mitophagy activation acts as the primary mechanism by which fisetin alleviates oxidative damage in D-gal-induced senescent GCs.

The AMPK/mTOR pathway, the central sensor of cellular energy and nutrient status, serves as a key switch regulating mitophagy activation and has been shown to be involved in the modulation of ovarian function [36]. For instance, AMPK/mTOR-mediated mitophagy has been reported to reduce ROS levels in oocytes and preserve mitochondrial function for ATP and estrogen synthesis to enhance the in vitro development of preantral follicles, and its activation also promotes steroid hormone synthesis and secretion as well as cumulus expansion in yak cumulus cells [37,38]. Emerging research has demonstrated that the pharmacological effects of fisetin against various diseases are mediated by upregulation of the AMPK/mTOR signaling pathway. Fisetin targeted AMPK and subsequently attenuated hepatic steatosis, modulated cholesterol metabolism and alleviated oxidative stress in a hypercholesterolemic mouse model [39]. In addition, fisetin injection increased autophagic activity and AMPK levels while inhibiting ER stress in pancreatic tissues, thereby effectively combating high-fat diet-induced nonalcoholic fatty pancreatic disease in mice [40]. In this study, our data revealed that fisetin exhibited strong binding affinity for the AMPK protein and that its supplementation upregulated the p-AMPK/AMPK ratio while downregulating the p-mTOR/mTOR ratio in D-gal-treated ovarian tissues in vivo and in cultured GCs in vitro, suggesting the involvement of the AMPK/mTOR signaling pathway in the protective effects of fisetin against D-gal-induced ovarian aging. Furthermore, addition of the selective AMPK inhibitor Compound C reversed the activating effect of fisetin on the AMPK/mTOR axis, leading to inhibition of mitophagy, aggravation of mitochondrial dysfunction, and exacerbation of oxidative stress in D-gal-induced senescent GCs, as evidenced by reduced LC3/TOMM20 co-localization, decreased MMP, and elevated ROS levels. Collectively, these findings suggest that fisetin prevents oxidative damage in follicular GCs by activating the AMPK/mTOR pathway to induce mitophagy, thereby supporting GC survival and delaying ovarian aging.

## 5. Conclusions

**Figure 9:**
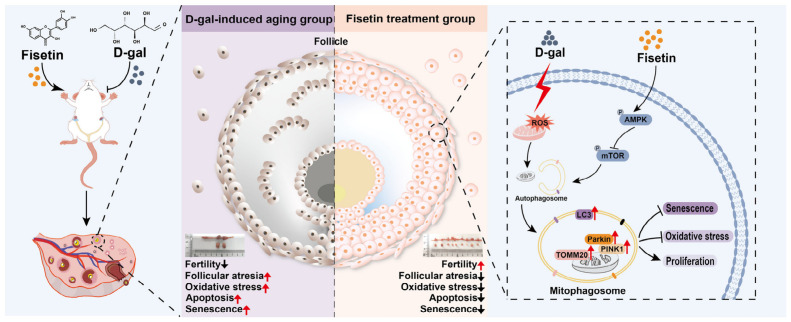
Fisetin attenuates ovarian aging by activating AMPK/mTOR-mediated mitophagy in D-gal-induced aging mice.

As shown in Figure 9, the existing data suggest that fisetin attenuates ovarian aging by restoring follicular GC function through activation of AMPK/mTOR-mediated mitophagy, thereby suppressing oxidative stress, alleviating mitochondrial dysfunction, reversing cell cycle arrest, and ultimately improving ovarian reserve. These findings suggest fisetin as a potential therapeutic agent for oxidative stress-related ovarian disorders and provide a candidate molecule for intervention in age-related fertility decline.

## Data Availability

The original contributions presented in this study are included in the article and Supplementary Materials. Further inquiries can be directed to the corresponding authors.

## Supplementary Materials

The following supporting information can be downloaded at: https://www.mdpi.com/article/10.3390/antiox15050602/s1, Table S1. Sequences of the PCR primers.

## References

[1] Zhang X., Zhang L., Xiang W. (2025). The impact of mitochondrial dysfunction on ovarian aging. J. Transl. Med..

[2] Begum I.A. (2025). Oxidative stress: Oocyte quality and infertility. Reprod. Toxicol..

[3] Dong J., Yang Z., Yuan Q., Zeng W., Mi Y., Zhang C. (2025). Preventive effect of fisetin on follicular granulosa cells senescence via attenuating oxidative stress and upregulating the wnt/β-catenin signaling pathway. Cells.

[4] Bucak M., Nazıroğlu M. (2026). Selenium protects granulosa cells from 3-nitropropionic acid-induced oxidative toxicity by regulating TRPM2-induced cell death. Reprod. Toxicol..

[5] Heng N., Hao H., Hu Y., Wang Y., Wang H., He W., Zhu N., Wang R., Xuan X., Zhu H. (2025). Metformin improves palmitate-induced follicular granulosa cell dysfunction by activating ULK1-mediated autophagy. Reprod. Sci..

[6] Zhou J., Guo M., Danzeng Q., Gu Q., Zhaxi M., Lu X., Deng J., Zhu L., Liu H. (2025). Lactate-HIF-1a axis promotes PINK1-dependent mitophagy to protect porcine granulosa cells against hypoxic stress. Int. J. Biol. Macromol..

[7] Xu G., Dong Y., Wang Z., Ding H., Wang J., Zhao J., Liu H., Lv W. (2023). Melatonin attenuates oxidative stress-induced apoptosis of bovine ovarian granulosa cells by promoting mitophagy via SIRT1/FoxO1 signaling pathway. Int. J. Mol. Sci..

[8] Zhu S., Tang M., Chen J., Li S., Xue R. (2025). Mitophagy protects against cisplatin-induced injury in granulosa cells. Toxics.

[9] El Sayed S., Saiyed D., Macri V.I., Asamoah-Mensah A., Segars J.H., Islam M.S. (2026). Beneficial effects of fisetin, a senotherapeutic compound, in women’s reproductive health and diseases: Evidence from in vitro to clinical studies. Nutrients.

[10] Ji X.M., Dong X.X., Li J.P., Tai G.J., Qiu S., Wei W., Silumbwe C.W., Damdinjav D., Otieno J.N., Li X.X. (2025). Fisetin clears senescent cells through the Pi3k-Akt-Bcl-2/Bcl-xl pathway to alleviate diabetic aortic aging. Phytother. Res..

[11] Murray K.O., Mahoney S.A., Ludwig K.R., Miyamoto-Ditmon J.H., VanDongen N.S., Banskota N., Herman A.B., Seals D.R., Mankowski R.T., Rossman M.J. (2025). Intermittent supplementation with fisetin improves physical function and decreases cellular senescence in skeletal muscle with aging: A comparison to genetic clearance of senescent cells and synthetic senolytic approaches. Aging Cell.

[12] Chahal S.K., Kabra A. (2024). Fisetin ameliorates polycystic ovary syndrome in rats via a mechanistic modulation of AMP-activated protein kinase and SIRT1 molecular pathway. Naunyn Schmiedebergs Arch. Pharmacol..

[13] Gao Q., Zhao D., He W. (2025). Protective effects of fisetin on ovarian ischemia-reperfusion injury in rats via modulation of the TLR4-MyD88-TRAF6 signaling pathway. Acta Cir. Bras..

[14] Ding H., Li Y., Chen S., Wen Y., Zhang S., Luo E., Li X., Zhong W., Zeng H. (2022). Fisetin ameliorates cognitive impairment by activating mitophagy and suppressing neuroinflammation in rats with sepsis-associated encephalopathy. CNS Neurosci. Ther..

[15] Deng Y., Jiang X., Che Z., Shang Y., Hu M., Wang W., Yu W., Yang B., Liu X. (2025). Fisetin carbon dots alleviate periodontitis by enhancing mitophagy through regulation of sirtuin 3 SUMOylation. J. Nanobiotechnol..

[16] Wang J., Li X., Li C., Liu L., Wang Z., Feng J. (2025). The Codonopsis pilosula water extract improves testicular inflammatory aging in D-galactose induced aging mice by modulating the CLEC7A/inflammasome pathway. J. Ethnopharmacol..

[17] Zhou G., Hu J., Xu M., Li Y., Chang R., Zeng J., Dan W., Peng L., Wang Z., Sun G. (2025). Honeybees fed D-galactose exhibit aging signs with changes in gut microbiota and metabolism. mSystems.

[18] Xie W., Deng L., Qian R., Huang X., Liu W., Tang S. (2024). Curculigoside attenuates endoplasmic reticulum stress-induced epithelial cell and fibroblast senescence by regulating the SIRT1-P300 signaling pathway. Antioxidants.

[19] de Magalhães J.P. (2025). An overview of contemporary theories of ageing. Nat. Cell Biol..

[20] Wang H. (2025). The role of granulosa cells in oocyte development and aging: Mechanisms and therapeutic opportunities. Semin. Cell Dev. Biol..

[21] Pandey A.N., Yadav P.K., Premkumar K.V., Tiwari M., Antony M.M., Pandey A.K., Chaube S.K. (2025). Damage mechanisms of bisphenols on the quality of mammalian oocytes. Hum. Reprod..

[22] Chauvin S. (2025). Role of granulosa cell dysfunction in women infertility associated with polycystic ovary syndrome and obesity. Biomolecules.

[23] Song H., Zhang R., Liu Y., Wu J., Fan W., Wu J., Liu Y., Lin J. (2025). Menstrual blood-derived endometrial stem cells ameliorate ovarian senescence by relieving oxidative stress-induced inflammation. Reprod. Sci..

[24] Wang Y.X., Zhang Y.S., Liu X., An J.Y., Li G.Y., Zou Y.X., Zheng Y.L., Fang Y., Li K.X., Zhu L. (2026). Zuogui pill ameliorates DNA damage and the senescence-associated secretory phenotype in ovarian stem cells to delay ovarian ageing through activation of SIRT1. Phytomedicine.

[25] Moustafa P.E., Abo El Nasr N.M.E., Shabana M.E., Saleh D.O. (2024). Fisetin mitigates letrozole-induced polycystic ovarian syndrome in rats: Crosstalk of AMPK/PI3K/AKT-mediated-Nrf2 antioxidant defense mechanism and the inflammasome NLRP3/NF-κB P65/IL-1β signaling pathways. Naunyn Schmiedebergs Arch. Pharmacol..

[26] Yang Z., Zhang J., Yuan Q., Wang X., Zeng W., Mi Y., Zhang C. (2024). Flavonoid fisetin alleviates ovarian aging of laying chickens by enhancing antioxidant capacity and glucose metabolic homeostasis. Antioxidants.

[27] Reagan-Shaw S., Nihal M., Ahmad N. (2008). Dose translation from animal to human studies revisited. FASEB J..

[28] Mayo Clinic AFFIRM: A Phase 2 Randomized, Placebo-Controlled Study of Alleviation by Fisetin of Frailty, Inflammation, and Related Measures in Older Women; ClinicalTrials.gov. 2018. https://clinicaltrials.gov/study/NCT03675724.

[29] Krishnakumar I.M., Jaja-Chimedza A., Joseph A., Balakrishnan A., Maliakel B., Swick A. (2022). Enhanced bioavailability and pharmacokinetics of a novel hybrid-hydrogel formulation of fisetin orally administered in healthy individuals: A randomised double-blinded comparative crossover study. J. Nutr. Sci..

[30] Park W.H. (2026). Oxidative assault: How pyrogallol’s pro-oxidant chemistry drives cell cycle arrest and apoptosis in cancer. J. Appl. Toxicol..

[31] Wei H.K., Qi J.J., Wang Y.Q., Qu H.X., Yan C.X., Li T.T., Wang Y., Sun H., Sun B.X., Liang S. (2025). Fisetin alleviates oxidative stress and promotes porcine early embryonic development via activation of the NRF2-ARE signalling pathway. Anim. Biosci..

[32] Yıldırım A.B., Göl M., Çimen L., Yiğin A., Amrein M., Küçükhüyük Ş. (2026). Effects of pirfenidone and fisetin on apoptosis and oxidative stress mechanisms in heart tissue of bleomycin-induced pulmonary fibrosis. J. Mol. Histol..

[33] Feng Q., He L., He Y., Li X., Xu R., Xu X., Jiang T., He Y. (2026). Fisetin ameliorates vascular calcification by regulating HNRNPA1-mediated ferroptosis. Ann. Vasc. Surg..

[34] Zeng X., Xie H., Zhang W., Yu Y., Wang M., Jiang Y., Guo R., Sun Y., Yang Q. (2026). Loss of PINK1 impairs mitophagy and accelerates ovarian aging independent of Parkin. Free Radic. Biol. Med..

[35] Song T., Yin F., Wang Z., Zhang H., Liu P., Guo Y., Tang Y., Zhang Z. (2023). Hsp70-Bim interaction facilitates mitophagy by recruiting parkin and TOMM20 into a complex. Cell Mol. Biol. Lett..

[36] Zhu X., Li H., Xue T., Wang S., Zhu R., Luo J., Ju R., Zhang P., Cui X., Jing X. (2025). Mechanistic study on the role of multi-pathway autophagy in ovarian aging: Literature review. Apoptosis.

[37] He J., Zhong Y., Li Y., Liu S., Pan X. (2025). Astaxanthin alleviates oxidative stress in mouse preantral follicles and enhances follicular development through the AMPK Signaling pathway. Int. J. Mol. Sci..

[38] Zhang R., Cui Y., Pan Y., Wang M., Yu S., Xu R., Ma W., Wang J., Zhong D., Jiao Z. (2025). CIRBP enhances the function of yak cumulus cells by activating AMPK/mTOR-mediated mitophagy. Biomolecules.

[39] Zhang Z., Chen X., Hu X., Liang J., Huang H., Lu W., Zhu M., Fang M., Yin L., Li W. (2026). Fisetin-loaded nanoparticles as a novel approach for cholesterol regulation in hypercholesterolemia: Targeting the ASGR1-mediated mTORC1/AMPK pathway. J. Nanobiotechnol..

[40] Sattari M., Karimpour A., Akhavan Taheri M., Larijani B., Meshkani R., Tabatabaei-Malazy O., Panahi G. (2025). Optimized effects of fisetin and hydroxychloroquine on ER Stress and autophagy in nonalcoholic fatty pancreas disease in mice. J. Diabetes Res..

