# Supplementary material for: Fisetin Attenuates D-Gal-Induced Ovarian Aging by Modulating Mitophagy via the AMPK/mTOR Pathway
Source: Antioxidants (Basel). 2026 May 9;15(5):602. doi: 10.3390/antiox15050602 (PMC13203114; doi:10.3390/antiox15050602)
Supplement: Supplementary file 1 [file antioxidants-15-00602-s001.zip › antioxidants-4296859-supplementary.pdf]

Supplementary data

Table S1. Sequences of the PCR primers

Gene name	Accession number	Primer sequence (5'-3')	Product size (bp)
Caspase-3	NM_001284409.1	AGCTTGGAACGGTACGCTAA<br>GAGTCCACTGACTTGCTCCC	117
PCNA	NM_011045.2	CCTGTGCAAAGAATGGGGTG<br>TCTCTATGGTTACCGCCTCC	114
CDK2	NM_016756.4	AGAAGATTGGAGAGGGCACG<br>ACACCTTCAGTCTCAGTGTCG	109
CDK6	NM_009873.3	TGCTGTGGAAGAAAAGTGCAGA<br>ACTCGCTGCCTTACACGC	218
Bax	NM_001411994.1	AAACTGGTGCTCAAGGCCC<br>GGTCCCGAAGTAGGAGAGGA	147
BCL2	NM_009741.5	GACTGAGTACCTGAACCGGC<br>AGTTCCACAAAGGCATCCCAG	72
Slc38a9	NM_178746.4	TGTGAAAGGGTCTTCGCCTC<br>GCCTGGAATCACCATCCACA	123
Telo2	NM_001163661.1	GTTATTGCACCCTGGCAAGC<br>GGCGCTTCACTCACAATCTC	172
Tsc2	NM_001403402.1	AGGCAGGTGCCATCACATAC<br>CAAGGGCAGGACTACTGTGG	116
Irs1	NM_010570.4	CCGGATACCGATGGCTTCTC<br>CCGCCACTTCTTCTCGTTCT	150
Wnt5a	NM_001256224.2	TTGTTGCTCCGGCCCAGAA<br>CCGGAACTGGTACTGGCATT	338
Deptor	NM_001037937.4	AAGAGTAGGCTAGGGGCCAT<br>TCCAGTGCAAAGGTCCCATC	372
Cp	NM_001276248.2	ACAAGGTCCGATTCTGTCTCC<br>CTGAGGTGTTAGCGGCTTGT	83
Atp6v1a	NM_001358203.1	AGTCGCGGAACCATTCACAT<br>GGGCTCGAAATGCAGAAAGT	203
Lepr	NM_001122899.2	CCTGGGCACAAGGACTGAAT<br>TGGACTGTTGGGAAGTTGGTA	101
Mapk1	NM_001038663.1	GCAAGGGAGAGATGGTGTAAG<br>CGTCACAGTGTCTAAGGGCT	181
β-actin	NM_007393.5	CACTGTCGAGTCGCGTCC<br>CGCAGCGATATCGTCATCCA	89

Caspase-3: cysteine-aspartic acid protease 3; *PCNA*: proliferating cell nuclear antigen; *CDK2*: cyclin-dependent kinase 2; *CDK6*: cyclin-dependent kinase 6; *Bax*: BCL2-associated X protein; *BCL2*: B cell lymphoma 2 ; *Slc38a9*: solute carrier family 38 member 9; *Telo2*: telomere maintenance 2; *Tsc2*: tuberous sclerosis complex 2; *Irs1*: insulin receptor substrate 1; *Wnt5a*: Wnt family member 5A; *Deptor*: DEP domain containing MTOR interacting protein; *Cp*: ceruloplasmin; *Atp6v1a*: ATPase, H^+ transporting, lysosomal V1 subunit A; *Lepr*: leptin receptor; *Mapk1*: mitogen-activated protein kinase 1.
